# Modulating excitation through plasticity at inhibitory synapses

**DOI:** 10.3389/fncel.2014.00093

**Published:** 2014-03-28

**Authors:** Vivien Chevaleyre, Rebecca Piskorowski

**Affiliations:** Centre National de la Recherche Scientifique, UMR8118, Université Paris DescartesParis, France

**Keywords:** inhibition, plasticity, PV+, CCK+, hippocampus

## Abstract

Learning is believed to depend on lasting changes in synaptic efficacy such as long-term potentiation and long-term depression. As a result, a profusion of studies has tried to elucidate the mechanisms underlying these forms of plasticity. Traditionally, experience-dependent changes at excitatory synapses were assumed to underlie learning and memory formation. However, with the relatively more recent investigation of inhibitory transmission, it had become evident that inhibitory synapses are not only plastic, but also provide an additional way to modulate excitatory transmission and the induction of plasticity at excitatory synapses. Thanks to recent technological advances, progress has been made in understanding synaptic transmission and plasticity from particular interneuron subtypes. In this review article, we will describe various forms of synaptic plasticity that have been ascribed to two fairly well characterized populations of interneurons in the hippocampus, those expressing cholecystokinin (CCK) and parvalbumin (PV). We will discuss the resulting changes in the strength and plasticity of excitatory transmission that occur in the local circuit as a result of the modulation of inhibitory transmission. We will focus on the hippocampus because this region has a relatively well-understood circuitry, numerous forms of activity-dependent plasticity and a multitude of identified interneuron subclasses.

## Different roles for different interneurons

It is commonly assumed that changes in inhibitory transmission will have consequences on synaptic plasticity at excitatory synapses. It has been known for over 30 years that pharmacological blockade of γ-Aminobutyric acid (GABA) receptors facilitates the induction of long-term potentiation (LTP) at excitatory synapses (eLTP), likely by increasing Ca^2+^ influx in the postsynaptic cell during the induction protocol (Wigstrom and Gustafsson, 1983). In addition, decreasing inhibition through long-term depression (LTD) at inhibitory synapses (iLTD) can also mediate a dis-inhibitory potentiation of excitatory drive (Ormond and Woodin, 2009).

Interneurons are classified according to several factors including axonal and dendritic connectivity, electrophysiological properties and expression of molecular markers. Based on these criteria, the hippocampus is one of the structures with the largest interneuron diversity (reviewed by Somogyi and Klausberger, 2005). In this mini-review, we will describe several forms of plasticity that have been ascribed to specific interneuron populations and discuss the resulting changes in the strength and plasticity at excitatory transmission. We will focus on hippocampal interneurons expressing cholecystokinin (CCK) and parvalbumin (PV). These two populations of interneurons are relatively well characterized in multiple brain areas (Freund and Katona, 2007; Armstrong and Soltesz, 2012) and recent studies have benefitted from genetic tools allowing their identification and modulation in hippocampal slices and *in vivo*. Our focus is restricted to studies performed in the hippocampus because the well-characterized circuitry has allowed for insight into how the numerous forms of plasticity expressed in inhibitory cells alters excitation and modulates network properties.

**Figure 1:**
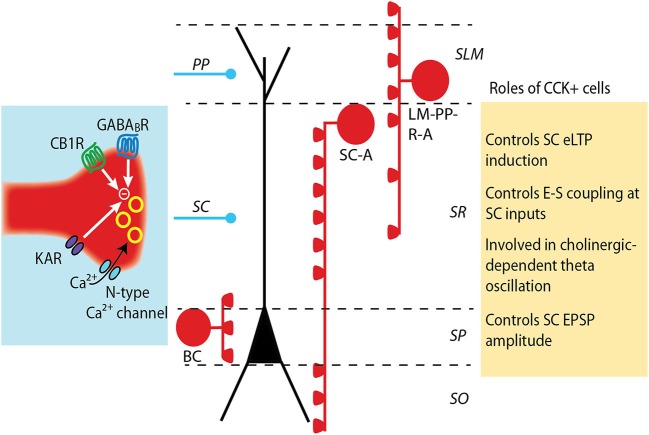
**Modulation and roles of GABA release from CCK+ interneurons.** CCK+ interneurons target pyramidal cell soma (basket cell, BS) or dendrites (Schaffer collateral-associated cell, SC-A and lacunosum moleculare-radiatum-perforant path-associated cell, LM-R-PP-A) (see Somogyi and Klausberger, 2005). The release of GABA from CCK+ cell terminals is mediated by N-type calcium channels, which provide a loose coupling between calcium influx and exocytosis and partially underlie the asynchronous release of GABA by these cells. GABA release is negatively controlled by the activation of several receptors: CB1 cannabinoid receptors, GABA_B_ receptors and kainate receptors. The decrease in GABA release differently impacts excitatory synapses depending on which subset of CCK+ interneuron synapses are depressed. A decrease in dendritic-targeting CCK+ synapse facilitates LTP induction at SC-CA1 synapses and increases the ability of an excitatory post synaptic potential (EPSP) to evoke an action potential (E-S coupling). When GABA release at somatic-targeting CCK+ synapses is depressed, a large increase in the amplitude of the SC EPSPs is observed, but distal perforant path (PP) EPSPs are unaltered.

**Figure 2:**
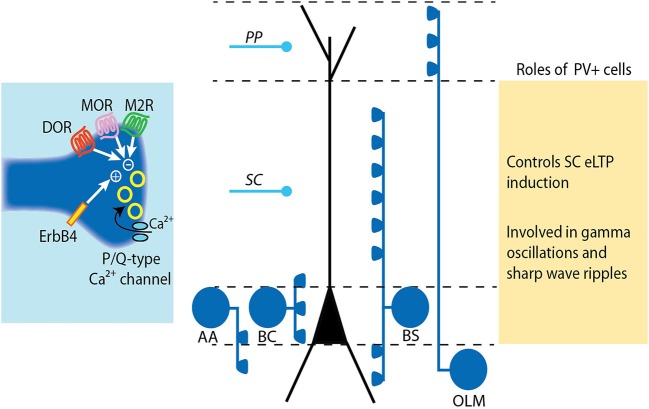
**Modulation and roles of GABA release from PV+ interneurons.** PV+ interneurons target either the soma (basket cell, BS), the axon (axo-axonic cell, AA) or the dendrites (bistratified cell, BS and oriens-lacunosum moleculare cell, OLM) of pyramidal cells (see Somogyi and Klausberger, 2005). The release of GABA from PV+ cell terminals is mediated by P/Q-type calcium channels. The tight coupling between calcium influx and exocytosis machinery results in precisely timed vesicle release. The release of GABA at PV+ cell synapses is negatively controlled by diverse receptors including mu- and delta-opioid receptors (MOR and DOR) and muscarinic M2 receptors. Conversely, GABA release from PV cells is increased by activation of the Neuregulin 1 receptor ErbB4. LTP induction at SC-CA1 synapses is impaired following ErB4 activation in PV+ cells due to increased pre-synaptic GABA release by PV+ cells.

CCK+ interneurons are considered to be highly plastic, as several neurotransmitters and neuromodulators have been revealed to alter synaptic transmission from these cells. PV+ interneurons, on the other hand, have been considered to be much more static, acting to control the firing frequency and timing of pyramidal cells. However, there is recent evidence that synaptic transmission from and onto PV+ interneurons can be plastic. We will briefly describe how GABA release from CCK+ and PV+ interneurons can be modulated, and discuss the consequences of these modulations on the excitatory plasticity and overall network function in the hippocampus (Figures 1, 2).

## Modulation of GABA release by CCK+ and PV+ interneurons

The kinetics of GABA vesicle fusion has been found to differ between CCK+ and PV+ interneurons. Axon terminals of CCK+ cells express N-type Ca^2+^ channels. These Ca^2+^ channels are loosely coupled to the Ca^2+^ sensor involved in vesicle fusion, thereby resulting in significant jittering and asynchronous release of GABA (Hefft and Jonas, 2005). Furthermore, different types of CCK+ interneurons, including basket cells, bistratified cells and trilaminar cells show asynchronous release (Daw et al., 2009). In contrast, PV+ interneuron axon terminals express P/Q-type Ca^2+^ channels, which are more tightly coupled to vesicle fusion because of their location in the active zone. As a consequence, PV+ interneurons have more synchronous release of GABA (Hefft and Jonas, 2005).

CCK itself, which is co-released with GABA, can modulate GABA release by both CCK+ and PV+ neurons (Földy et al., 2007). In CCK+ cells, CCK release activates CCK2 receptors on pyramidal cells, resulting in retrograde endocannabinoid release and pre-synaptic activation of cannabinoid type 1 receptors (CB1R) and reducing GABA release (Földy et al., 2007; Karson et al., 2008). In contrast, activation of CCK2 receptors on PV+ basket cells results in the activation of a pertussis-toxin sensitive G-protein (Gi/o) coupled pathway that results in intracellular calcium release, transient receptor potential (TRP) channel activation and membrane depolarization (Lee et al., 2011). This membrane depolarization results in increased GABA release. Interestingly, PV+ bi-stratified cells showed no response to CCK, indicating that this modulation is specific to somatic inhibition and may be an important complementary component to the CCK+ cell modulation by CCK.

GABA release by CCK+ cells is uniquely altered by several modulators. For instance, the synchronous release of GABA can be decreased by presynaptic kainate receptors (Daw et al., 2010). In addition, the GABA_B_ receptor is detected in CCK+ cells but not in PV+ interneurons (Sloviter et al., 1999) and there is experimental evidence suggesting that activation of these receptors powerfully decreases GABA release from CCK+ cells (Neu et al., 2007). Furthermore, CCK+ cells are likely the only class of interneurons expressing CB1R (Marsicano and Lutz, 1999; Takács et al., 2014).

The exclusive modulation of one type of interneuron can have interesting functional consequences. For example, in fast-spiking PV+ basket cells, mu-opioid receptor activation hyperpolarizes the membrane and depresses GABA release while nearby CCK+ basket cells are unaffected by the mu-opioid receptor activation but are uniquely modulated by cannabinoid application (Glickfeld et al., 2008). Likewise, a comparison of the action of acetylcholine on different soma-targeting PV+ and CCK+ basket cells revealed that GABA release was diminished by M2-type muscarinic receptor activation uniquely in PV+ cells whereas CCK+ cell transmission was inhibited via cannabinoid signaling (Szabó et al., 2010). Thus, even though these interneuron classes receive similar inputs and have similar axonal arbors, their properties endow them with very different frequency tuning properties and are likely active at different times (Glickfeld and Scanziani, 2006). Furthermore, the distinct modulation of these different perisomatic interneurons can act to shift pyramidal cells into different modes of integration.

## Consequences of CCK+ interneuron plasticity on excitatory cell transmission

The CB1R is one of the most highly expressed G protein-coupled receptors in the nervous system (Herkenham et al., 1990). These receptors are involved in the action of endogenous cannabinoids (eCBs), which are synthetized from membrane lipid precursors by the postsynaptic cell and act as retrograde messengers to depress transmitter release from presynaptic terminals (for a general review, see Chevaleyre et al., 2006). All of the CB1-dependant plasticity discussed below are known to occur in CCK+ interneurons; however, it should be noted that not all CCK+ interneurons express CB1 receptors.

In the hippocampus, eCBs are involved in two forms of synaptic plasticity. When transiently released, for instance by depolarization of the postsynaptic cell, they mediate a short-term (~1 min) depression of GABA release, a phenomenon called depolarization-induced suppression of inhibition (DSI). This phenomenon was initially described more than 20 years ago in the cerebellum (Llano et al., 1991) and hippocampus (Pitler and Alger, 1992). The retrograde action of eCBs was attributed by Wilson and Nicoll (2001). The fast onset of DSI and the lack of sensitivity of tetrodotoxin (TTX) -resistant miniature IPSCs to DSI (Alger et al., 1996) are in agreement with a direct block of N-type Ca^2+^ channels by β/γ subunits of the G protein, an effect that was initially demonstrated in expression systems (Mackie and Hille, 1992).

When a more sustained release of eCB is evoked, for instance following activation of group I metabotropic glutamate receptor (mGluR-I), eCBs can mediate a long-term depression of inhibitory transmission. Several minutes of CB1R activation are needed for a lasting depression to be induced. This more sustained CB1R activation probably allows for significant changes in second messenger and phosphorylation levels of downstream target molecules. Consistently, protein kinase A (PKA) activity and the active zone proteins RIM1α and Rab3b are needed for iLTD induction (Chevaleyre et al., 2007; Tsetsenis et al., 2011), indicating that iLTD results in a change on the release machinery.

Because of the specific expression of CB1R in CCK+ cells, eCB-mediated plasticity initially offered a useful tool to dissect out the role of CCK+ cells in controlling excitatory transmission. Several studies reported that the decrease in GABA release from CCK+ cells could facilitate LTP induction at the Schaffer collateral (SC) -CA1 excitatory synapse. Not surprisingly, the time course of the facilitation follows the time course of the eCB-mediated plasticity. For instance, it was initially described that the dis-inhibition occurring during DSI provides a transient facilitation of LTP induction at excitatory synapses (Carlson et al., 2002). In contrast, induction of iLTD by eCB provides a long-lasting facilitation on the induction of eLTP (Chevaleyre and Castillo, 2004; Zhu and Lovinger, 2007). The spatial localization of the facilitation depends on the induction protocol used to evoke eCB release. DSI is a single-cell phenomenon, thus eLTP facilitation will only occur onto the cell expressing DSI. However, DSI targets multiple CB1R-sensitive inhibitory synapses along the somato-dendritic compartment, and will likely facilitate LTP induction at excitatory inputs targeting different locations of the apical dendrite. In contrast, iLTD can be evoked by a very localized activation of the Schaffer collaterals. The activation of mGluR-I onto pyramidal neurons triggers eCB release that hetero-synaptically decreases GABA release from nearby inhibitory terminals (Chevaleyre and Castillo, 2003). Because iLTD is spatially restricted to the region surrounding the stimulated excitatory fibers, eLTP facilitation is limited to the nearby dendritic region (Chevaleyre and Castillo, 2004). However, it was recently reported that iLTD can be evoked with repetitive postsynaptic firing, indiscriminately affecting somatic and dentritic inhibitory inputs (Younts et al., 2013). Therefore, it is expected that facilitation of eLTP will not be spatially restricted following this mode of induction.

Independently of the facilitation of eLTP described above, the decrease in GABA release from CCK+ cells can also increase the ability of an excitatory post synaptic potential (EPSP) to evoke an action potential (E-S coupling) and directly increase the size of the EPSP at the SC-CA1 synapse. The first effect was observed after inducing iLTD with synaptic activity of the SC inputs or with repetitive postsynaptic depolarization. Action potential firing was extracellularly monitored (Chevaleyre and Castillo, 2003) or recorded in individual pyramidal cells (Younts et al., 2013), and was increased with both iLTD inducing protocols. The second effect, i.e., a direct increase in the amplitude of SC EPSP, was reported recently by two studies using a paired stimulation between proximal (SC) and distal perforant path (PP) excitatory inputs, termed input-timing dependent plasticity (ITDP; Dudman et al., 2007). The initial study showed that the pairing protocol induced a potentiation of SC-EPSPs, and that this potentiation is dependent on eCB-mediated LTD at inhibitory synapses (Xu et al., 2012). The dependence on CB1R strongly suggests that the interneurons expressing iLTD were CCK+ cells. This idea was formally demonstrated in a second thorough and elegant study using multiple techniques to better elucidate the phenomenon (Basu et al., 2013). The authors showed that transmission from CCK+ interneurons is depressed following the ITDP protocol. In addition, this depression concerns perisomatic CCK+ terminals and is mediated by eCB release during the ITDP protocol. Finally, they showed that most of the increase in EPSP amplitude following the ITDP protocol is the result of the eCB-mediated iLTD at CCK+ terminals. These studies convincingly show that CCK+ interneurons targeting the soma of pyramidal neurons are playing an important role in controlling the strength of SC inputs. These data therefore suggest that CCK+ interneurons should contribute significantly to the feed-forward (FF) inhibition evoked by SC stimulation. Indeed, using optogenetics to silence CCK+ interneurons, the authors show that CCK+ cells mediate a major proportion of the FF inhibition elicited by SC stimulation, and that silencing transmission from CCK+ cells induced a large increase in SC-mediated EPSPs.

Altogether, these studies reveal a dual role of CCK+ interneurons in the control of excitatory transmission and plasticity. While a decrease in GABA release from dendritic-targeting CCK+ cells can facilitate LTP induction at SC-CA1 excitatory synapses, a decrease in GABA release from somatic-targeting CCK+ basket cells will directly increase the amplitude of the EPSPs. These studies highlight the importance of determining the subclass of interneuron by using a combination of protein markers, physiological properties and dendritic and axonal arborizations.

## Consequences of PV+ interneuron modulation on excitatory cell transmission

Excitatory synapses onto interneurons are known to express either LTP or LTD via activation of calcium-permeable glutamate receptors (Kullmann and Lamsa, 2007). Cell-type specific rules have been identified in a study examining five common interneuron subtypes, as defined by axonal projections and molecular expression profiles (Nissen et al., 2010). In this work, the authors found that excitatory synapses express LTP onto PV+ basket cells and LTD onto bistratified cells. Both of these phenomena were independent of N-Methyl-D-aspartate (NMDA) receptor activation and potentially act to shift the inhibition on excitatory cells from the dendrites to the soma. A closer examination of the FF and feedback (FB) excitatory inputs onto PV+ basket cells found that NMDA receptors are only found at synapses with FB afferents, leading to a narrower frequency tuning of LTP at these inputs than at FF inputs (Le Roux et al., 2013). Given the importance of FF inhibition to ensure the temporal fidelity of pyramidal cell firing (Pouille and Scanziani, 2001) and the very tight time-lock of basket cell interneurons and pyramidal cells during sharp wave ripples (Klausberger and Somogyi, 2008), it is possible the different properties of LTP at FF and FB synapses is permitting PV+ cells to modulate their activity in accordance with excitation.

PV+ basket cells in area CA1 have recently been shown to undergo a long-term increase in excitability in response to brief high frequency stimulation of SC inputs (Campanac et al., 2013). It was elegantly shown that this enhanced FF inhibition in area CA1 was due to an increase in the inherent excitability of PV+ cells resulting from activation of mGluR5 and subsequent down-regulation of D-type potassium current carried by Kv1 channels, termed LTP-IE_*PV−BC*_. The authors demonstrated that clustered spiking in the γ-range was increased, allowing for the speculation that this plasticity may provide a use-dependent modulation of hippocampal γ-oscillations, or even potentially allow for a modulation of the phase lag of PV+ basket cells during θ-activity.

PV+ interneurons may be playing an interesting role in mediating the ability of CA3 neurons to excite CA2 pyramidal cells. A very strong FF inhibition at the SC-CA2 synapse induces a very large hyperpolarization in CA2 pyramidal neurons and completely prevents SC axons from driving firing in CA2 pyramidal neurons (Chevaleyre and Siegelbaum, 2010; Kohara et al., 2014). Upon closer examination, this inhibition was found to undergo an iLTD in response to 10, θ-burst, and 100 Hz stimulus protocols. Furthermore, this iLTD was mediated entirely by the activation of delta opioid receptors, resulting in a lasting decrease in GABA release. Furthermore, by using optogenetics to elicit an IPSC from PV+ interneuron terminals, it was demonstrated that PV+ interneurons are responsible for this plasticity (Piskorowski and Chevaleyre, 2013). Given that CA2 pyramidal neurons express multiple factors that inhibit post-synaptic LTP at the SC-CA2 synapse (Zhao et al., 2007; Simons et al., 2009; Lee et al., 2010), this pre-synaptic iLTD in PV+ cells may be the major mechanism by which the excitability of the SC-CA2 synapse is modulated.

Changes in PV+ cell plasticity during development is thought to underlie the “critical periods” in cortical development when neural circuits undergo large adaptations in response to the environment (see review by Takesian and Hensch, 2013). Interestingly, there is growing evidence that PV+ cells in adult hippocampal circuits are modulated by similar mechanisms during learning.

This premise is supported by the finding that the trophic factor neuregulin1 (NRG1), which is a critical element in PV+ maturation during development, acts on adult PV+ cells to increase GABA transmission, resulting in a suppression of LTP induction at SC-CA1 synapses (Pitcher et al., 2008; Chen et al., 2010). When the NRG1 receptor, ErbB4, was selectively knocked out from PV+ cells, LTP at SC-CA1 synapses was increased and no longer repressed by NRG1. Interestingly, the PV+ cell specific ErbB4 knockout animals display a deficit in contextual fear conditioning, revealing an important role of PV+ cells in hippocampal learning (Chen et al., 2010). A recent and compelling study by Donato et al. (2013) has shown that PV+ basket cells in area CA3 show a change in activity state following contextual fear conditioning or environmental enrichment, two treatments found to respectively decrease or improve performance of hippocampal-dependent novel object recognition. The intensity of PV staining at axonal terminals was used as an indicator of PV+ cell activity state: high PV levels in the non-plastic state and low PV levels in the highly-plastic state. These observations are consistent with previous reports that pre-synaptic PV levels are able to modulate pre-synaptic calcium levels and GABA release in cerebellar interneurons during development (Collin et al., 2005). Furthermore, manipulations of the perineuronal net, an extracellular matrix that grows and shrinks during developmental “critical periods” and releases NRG1 and other PV+ cell modulators, can reset the PV+ cells to a highly-plastic low PV condition in the adult hippocampus (Donato et al., 2013) indicating that additional factors regulating PV+ cell excitability during development may control plasticity in the adult.

## Perspective

At the circuit level, a recent study reported that cholinergically-driven θ-oscillations in CA1 involves an inhibitory circuit consisting mainly of CCK+ interneurons (Nagode et al., 2014). This conclusion was based on the observation that cholinergically-driven oscillatory IPSCs were sensitive to cannabinoids and optogenetic silencing of CCK+ cells, but not PV+ cells. Thus, while CCK+ cells may be involved in low frequency oscillations such as θ-rhythm, PV+ cells may play a more prominent role in faster rhythms such as γ and sharp wave ripple oscillations. However, removing inhibition onto PV+ interneurons also affects θ-oscillation *in vivo* in CA1, suggesting a complex interaction between different interneuron types in oscillatory activity (Wulff et al., 2009). With the high diversity of PV+ interneurons in the hippocampus, there is also a very large level of diversity of synaptic plasticity. Recordings performed *in vivo* that take into account the axonal arbors and cell-type specific markers of interneurons, have revealed that each cell type has a potential role in the network activity of the hippocampus during a specific behavior (for example, Klausberger et al., 2003, 2004; Tukker et al., 2007; Lapray et al., 2012). Distinct differences in PV+ cell projection patterns and activities have been found in different hippocampal regions (Tukker et al., 2013). Even with all of this complexity, *in vivo* studies in which transmission from all PV+ cells has been removed reveal very interesting changes in behavior and hippocampal network activity (Korotkova et al., 2010; Murray et al., 2011; Royer et al., 2012). Deciphering how each subclass of interneuron dynamically contributes to network function during learning and disease states is a worthy goal for future work.

## Conflict of interest statement

The authors declare that the research was conducted in the absence of any commercial or financial relationships that could be construed as a potential conflict of interest.
